# Symmetric dimethylation of poly-GR correlates with disease duration in *C9orf72* FTLD and ALS and reduces poly-GR phase separation and toxicity

**DOI:** 10.1007/s00401-019-02104-x

**Published:** 2019-12-12

**Authors:** Lauren M. Gittings, Steven Boeynaems, Daniel Lightwood, Alison Clargo, Sarfaraj Topia, Lisa Nakayama, Claire Troakes, David M. A. Mann, Aaron D. Gitler, Tammaryn Lashley, Adrian M. Isaacs

**Affiliations:** 1 Department of Neurodegenerative Disease, UCL Queen Square Institute of Neurology, London, WC1N 3BG UK; 2 UK Dementia Research Institute at UCL, Cruciform Building, Gower Street, London, WC1E 6BT UK; 3 Queen Square Brain Bank for Neurological Disorders, UCL Queen Square Institute of Neurology, London, UK; 4 Department of Genetics, Stanford University School of Medicine, Stanford, CA USA; 5 UCB Pharma, 216 Bath Road, Slough, SL1 3WE UK; 6 London Neurodegenerative Diseases Brain Bank, Institute of Psychiatry, Psychology and Neuroscience, King’s College London, London, UK; 7 Division of Neuroscience and Experimental Psychology, School of Biological Sciences, University of Manchester, Salford Royal Hospital, Salford, UK

A GGGGCC repeat expansion in *C9orf72* is the most common genetic cause of frontotemporal dementia (FTD) and amyotrophic lateral sclerosis (ALS). Pathologically, patients are characterised by TDP-43 pathology and distinct inclusions containing dipeptide repeat proteins (DPRs) that are produced by repeat associated non-ATG initiated translation of the repeat expansion. This produces five different DPRs: poly-GA, poly-GR poly-PR poly-AP and poly-GP. Poly-GR and poly-PR have been shown to be highly toxic in in vitro and in vivo models, but the mechanisms are not entirely clear [1]. We investigated whether methylation of arginine residues in poly-GR (which is much more abundant than poly-PR) contributes to disease pathogenesis. Three types of arginine methylation can occur, monomethylarginine (MMA), or dimethylarginine in a symmetric (SDMA) or asymmetric (ADMA) confirmation. ADMA is the most prevalent modification with MMA and SDMA occurring at approximately 20–50% that of ADMA [2]. The importance of arginine methylation in FTD and ALS has recently come to light as methylation of arginine residues within the FTD/ALS-linked proteins FUS and hnRNPA2 is an important regulator of their liquid–liquid phase transition [7].

The presence of methylated DPRs has so far been suggested through indirect methods [3, 5, 10]. Therefore, in order to investigate DPR arginine methylation in *C9orf72* FTD/ALS we generated and characterised two novel antibodies that detect the two forms of dimethylated poly-GR (Supplementary Fig. 1, online resource). In frontal cortex, they stained cytoplasmic inclusions only in *C9orf72* cases and did not label TDP-43 inclusions (Supplementary Figs. 2 and 3, online resource). A comparison to p62 inclusion numbers in the 15 *C9orf72* cases available at the Queen Square Brain Bank showed that 48% and 7% of p62 inclusions were positive for ADMA-GR and SDMA-GR respectively. These data show that arginine methylation of poly-GR is a common post-translational modification in *C9orf72* patient brain. Interestingly, a rare *C9orf72* homozygous case had few SDMA-GR inclusions (Supplementary Fig. 4, online resource), despite a high load of ADMA-GR, abundant DPR protein pathology and severe clinical features [6]. Given the early age of disease onset and short disease duration in the homozygous case, in conjunction with a low SDMA-GR burden, we hypothesised that SDMA-GR may correlate with clinical severity. To investigate this, the number of ADMA-GR and SDMA-GR inclusions were quantified in the frontal cortex of 37 *C9orf72* cases (*C9orf72*-FTLD *n* = 22, *C9orf72*-ALS *n* = 15; details in Supplementary Table 1) and correlated with age at disease onset, age at death, disease duration and post-mortem delay (Supplementary Table 2). This identified a positive correlation between SDMA-GR and disease duration (*r* = 0.5139, *p* = 0.0026) (Fig. 1a), and age at death (*r* = 0.4568, *p* = 0.0045) (Fig. 1b). The correlations remained significant when normalised to total DPR burden, measured by p62 staining, (Fig. 1c, d) (Supplementary Table 3), ruling out that SDMA-GR levels were simply reflecting total DPR levels. One possible interpretation of our finding that greater numbers of SDMA-GR are correlated with a longer disease duration and later age at death is that SDMA-GR is protective.

**Fig. 1:**
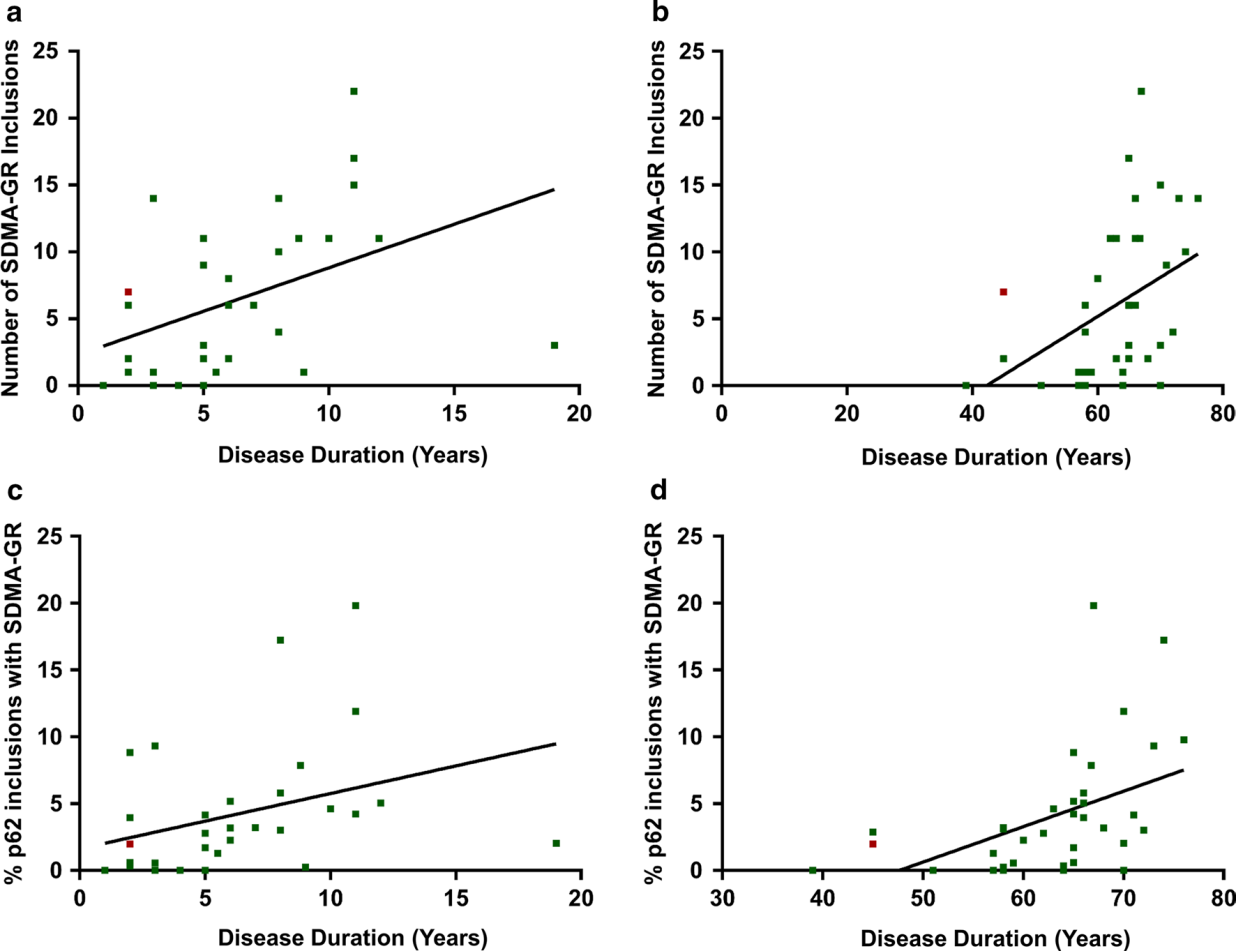
SDMA-GR correlates positively with disease duration and age at death in *C9orf72* patients. Individual *C9orf72* cases are represented by green squares and the homozygous case as a red square. Positive correlation between the number of SDMA-GR inclusions and **a** disease duration (*r* = 0.51, *p* = 0.0026, *n* = 32), and **b** age at death (*r* = 0.46, *p* = 0.0045, *n* = 37). Positive correlation between the percentage of p62 inclusions with SDMA-GR and **c** disease duration (*r* = 0.47, *p* = 0.0061, *n* = 32) and **d** age at death (*r* = 0.55, *p* = 0.0005, *n* = 37). Spearman’s rank correlation coefficient performed for all analyses

We therefore investigated the effect of methylation on GR phase separation and toxicity. We generated synthetic (GR)_20_ peptides with or without ADMA/SDMA-modifications (Fig. 2a). Both ADMA- and SDMA-poly-GR displayed reduced phase separation, as evident from the higher saturation concentration assayed by turbidity measurements (Fig. 2b, c). Imaging showed that while droplets were less abundant for dimethylated poly-GR, they were larger in size (Fig. 2d). As we have previously reported for other basic peptides, such differences in droplet size stem from differences in the interaction strength of the phase separating molecules [4]. Increased interaction strength increases droplet viscosity and surface tension, thereby reducing droplet fusion, leading to smaller droplets. Increased interaction strength will also lower the saturation concentration of phase separation. Hence, both turbidity measurements and droplet imaging point to a decrease in interaction strength between dimethylated poly-GR molecules. We next compared toxicity to primary neuronal cultures and found that dimethylated poly-GR was less toxic than unmethylated poly-GR (Fig. 2e–g), even though uptake of the peptides was similar (Supplementary Fig. 5, online resource). Hence, the same post-translational modifications that reduce poly-GR phase separation also reduces its neuronal toxicity.

**Fig. 2:**
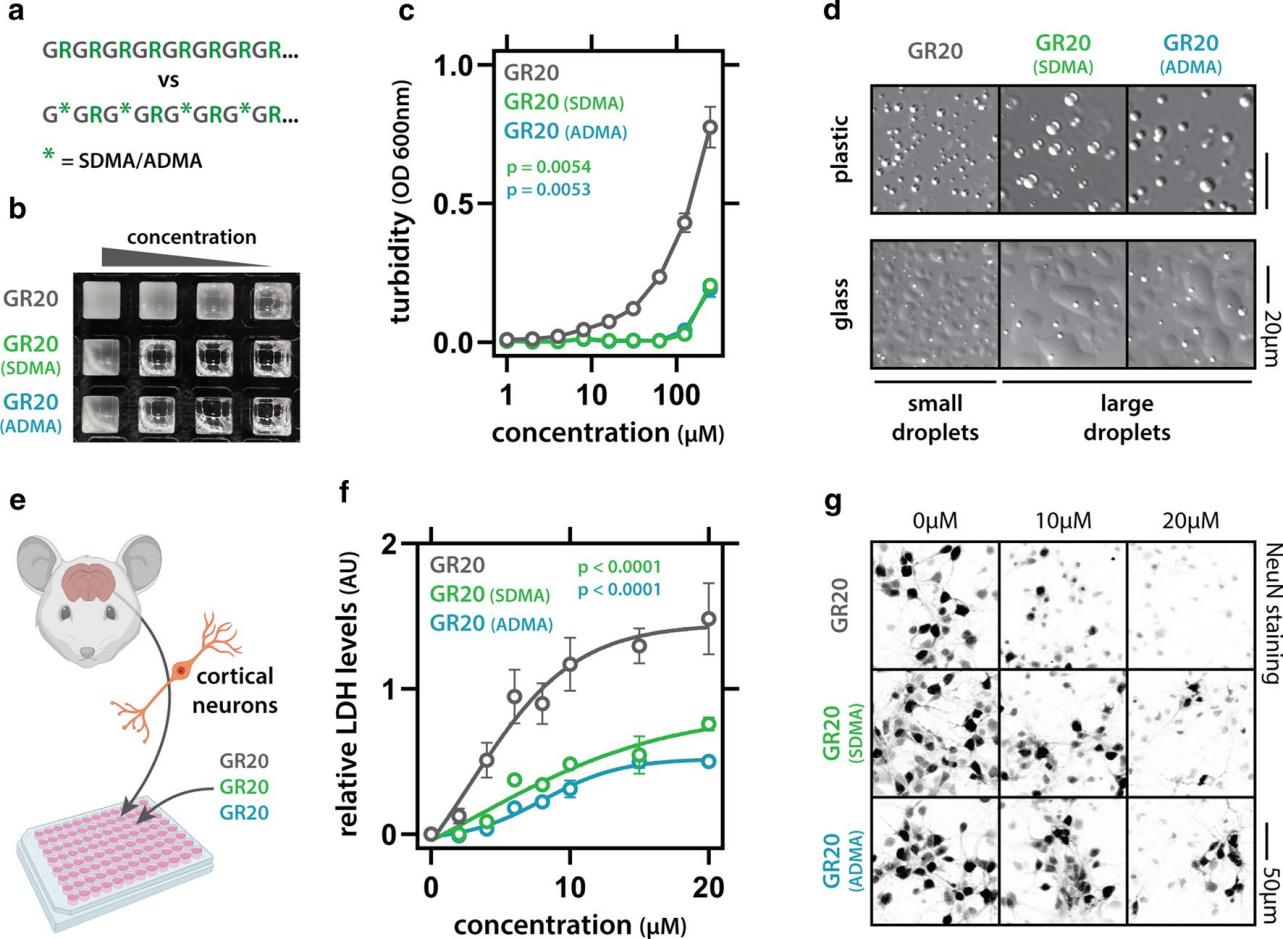
Arginine dimethylation reduces phase separation and neurotoxicity of poly-GR. **a** Scheme showing methylation of synthetic (GR)_20_ peptides. **b** Phase separation of (GR)_20_ is reduced by SDMA and ADMA modification, as seen by a reduction in the concentration-dependent turbidity increase, quantified in (**c**). Mean (*n* = 3) and SEM are shown. Two-way ANOVA. **d** Pictures showing increased droplet size of dimethylated (GR)_20_. Concentration 250 µM. **e** Scheme of the neurotoxicity assay setup. **f** Quantification of exogenously added (GR)_20_ toxicity to mouse primary cortical neurons. Mean (*n* = 4) and SEM are shown. Two-way ANOVA. **g** Pictures showing loss of neurons in (GR)_20_-treated cultures (NeuN staining)

A previous report found no correlation of poly-GR with neurodegeneration or clinical phenotypes [8], while two more recent studies showed poly-GR inclusions did correlate with neurodegeneration [9, 10]. As these previous studies did not specifically label SDMA-GR, our findings provide new insights into the relationship of poly-GR inclusions with clinical phenotypes. Future studies are required to investigate why only SDMA-GR is associated with longer disease duration and age at death, and not ADMA-GR, as both forms were able to affect phase separation and toxicity (see Supplementary discussion, online resource for further discussion). In summary, our data show that arginine methylation is a common post-translation modification of poly-GR in *C9orf72* patient brain that may influence disease course.

## Electronic supplementary material

Below is the link to the electronic supplementary material.

Supplementary file1 (PDF 807 kb)
