# Supplementary material for: Symmetric dimethylation of poly-GR correlates with disease duration in C9orf72 FTLD and ALS and reduces poly-GR phase separation and toxicity
Source: Acta Neuropathol. 2019 Dec 12;139(2):407–10. doi: 10.1007/s00401-019-02104-x (PMC6989575; doi:10.1007/s00401-019-02104-x)
Supplement: Supplementary file 1 — Supplementary file1 (PDF 807 kb) [file 401_2019_2104_MOESM1_ESM.pdf]

Symmetric dimethylation of poly-GR correlates with disease duration in *C9orf72* FTLD and ALS and reduces poly-GR phase separation and toxicity

Lauren M. Gittings, Steven Boeynaems, Daniel Lightwood, Alison Clargo, Sarfaraj Topia, Lisa Nakayama, Claire Troakes, David M.A. Mann, Aaron D. Gitler, Tammaryn Lashley, Adrian M. Isaacs

Supplementary Material

Competing interests

DL, AC and ST are paid employees of UCB. DL holds stocks in UCB Pharma. All other authors declare that they have no conflict of interest.

Funding

LG is funded by the Leonard Wolfson Centre for Experimental Neurology. SB acknowledges a long-term fellowship from EMBO. ADG is supported by the NIH (grant number NS097263). TL is supported by an Alzheimer's Research UK Senior Fellowship and the Leonard Wolfson Centre for Experimental Neurology. AI receives funding from the European Research Council (ERC) under the European Union's Horizon 2020 research and innovation programme (648716 - C9ND), Alzheimer's Research UK, the Motor Neurone Disease Association and the UK Dementia Research Institute which receives its funding from DRI Ltd, funded by the UK Medical Research Council, Alzheimer's Society and Alzheimer's Research UK. The Queen Square Brain Bank for Neurological Disorders is supported by the Reta Lila Weston Institute for Neurological Studies and the Medical Research Council. The MRC London Neurodegenerative Diseases Brain Bank receives funding from the UK Medical Research Council (MR/L016397/1). The MRC London Neurodegenerative Diseases Brain Bank and Manchester Brain Bank are part of the Brains for Dementia Research programme, jointly funded by Alzheimer's Research UK and Alzheimer's Society.

Authors' contributions

LG, SB, TL and AI designed the experiments and wrote the manuscript; LG performed immunostaining, imaging and image analysis; SB, LN, and ADG designed and performed the phase separation and primary neuron experiments; DL, AC and ST generated the methyl-GR antibodies; TL, CT and DM provided tissue sections. All authors read and approved the final manuscript.

Acknowledgements

The authors would like to thank the Queen Square Brain Bank for Neurological Disorders, UCL Institute of Neurology, London, UK; the MRC London Neurodegenerative Diseases Brain Bank, Institute of Psychiatry, Psychology and Neuroscience, King's College London, UK; and the Manchester Brain Bank; University of Manchester, Manchester, UK for providing tissue used in this study.

Materials and methods

Antibody generation

Rabbit monoclonal ADMA-GR and SDMA-GR antibodies were generated by UCB Pharma. Two separate rabbits were immunised with peptide N-acetyl-GRGRGRGR-NH_2 modified with either asymmetric dimethyl arginine (ADMA-GR) or symmetric dimethyl arginine (SMDA-GR) (Peptide Protein Research Ltd) respectively. Immunisations were also performed with ADMA-PR and SDMA-PR peptides but no specific antibodies were generated. Immunisations were performed using the peptides conjugated to KLH, OVA and BSA in 3 successive sub-cutaneous administrations with the first dose being given in complete Freund's adjuvant and subsequent shots provided in incomplete Freund's adjuvant with two-week gaps between immunisations. Termination occurred 14 days after the final boost with single cell suspensions of spleen, blood, lymph node and bone marrow prepared and frozen in 10 % dimethyl sulfoxide (DMSO) in fetal calf serum (FCS) at -80 °C.

B cell culture screening was performed using a method previously reported [20]. Briefly, the presence of peptide-binding antibodies in B cell culture supernatants was determined using a homogeneous fluorescence-based binding assay using Superavidin beads (Bangs Laboratories) coated with either biotinylated ADMA-GR or SDMA-GR. Binding was revealed with a goat anti-rabbit IgG Fcγ-specific Cy-5 conjugate (Jackson ImmunoResearch). Plates were read on a Mirrorball fluorescence cytometer (TTP Labtech).

Following primary screening, positive supernatants were consolidated onto 96-well bar-coded master plates then screened for binding against ADMA-GR, SDMA-GR, unmodified-GR peptide, as well as unmodified N-acetyl-PRPRPRPR-NH2 peptide (PR), and PR peptide modified with either asymmetric dimethyl arginine (ADMA-PR) or symmetric dimethyl arginine (SMDA-PR) (Peptide Protein Research Ltd). In addition, an irrelevant peptide from an unrelated protein was used as a negative control. Binding assays involved an ELISA using a streptavidin capture step. The ELISA assay involved coating 384-well Maxisorp ELISA plates (Thermo Fisher Scientific) with Streptavidin at 2 µg/ml in a carbonate coating buffer (dH_2O, + 0.16 % Na_2CO_3, + 0.3 % $NaHCO_3$) before biotinylated peptide was added at 2 µg/ml. Plates were blocked with 1 % bovine serum albumin (BSA) in phosphate buffered saline (PBS) and then incubated with 10 µl/well of B cell culture supernatant. Plates were then incubated with a secondary HRP-conjugated goat anti-rabbit IgG Fc-specific antibody (1:5000; Jackson ImmunoResearch) and visualisation of binding was revealed using 3,3',5,5'-Tetramethylbenzidine substrate (TMB; Millipore). The optical density was measured at 630 nM using Synergy 2 microplate reader (BioTek).

B cell supernatants demonstrating specificity to the ADMA-GR or SDMA-GR peptides were selected for variable region recovery and cloning as previously described [8], utilising a fluorescent foci technique using beads coated with biotinylated peptide to identify and isolate antigen-specific B cells from positive culture wells. Specific antibody variable region genes were recovered from single B cells by reverse transcription (RT)-PCR using heavy and light chain variable region-specific primers. PCR primers contained restriction sites at the 3' and 5' ends allowing cloning of the variable region into a rabbit IgG1

(VH) or rabbit kappa (VL) mammalian expression vector. Heavy and light chain constructs were co-transfected into HEK-293 cells using 293fectin transfection reagent (Invitrogen). After 7 days of recombinant antibody expression, the supernatants were harvested and antibodies were re-screened for selectivity using the specificity assays described above. IgG were purified on an AKta system (GE healthcare) using affinity chromatography (protein A) followed by size exclusion (SE)-HPLC to produce a product >98% pure monomer species.

Cases

Tissue from *C9orf72* cases used in this study was obtained from the Queen Square Brain Bank (QSBB) for Neurological Disorders, UCL Queen Square Institute of Neurology, University College London (*C9orf72*-FTLD, n = 13; *C9orf72*-ALS, n = 2); the MRC London Neurodegenerative Diseases Brain Bank, Institute of Psychiatry, Psychology and Neuroscience, King's College London (*C9orf72*-ALS, n = 8); and the Manchester Brain Bank; University of Manchester (*C9orf72*-FTLD, n = 7; *C9orf72*-ALS, n = 5). All cases had previously had *C9orf72* neuropathology confirmed. The mean age at onset, age at death and disease duration for the different groupings of *C9orf72* patients analysed in this study are shown in Table 1. Non-*C9orf72* FTLD-TDP A (n=3) and neurologically normal control cases (n=3) were obtained from QSBB. Details of each individual case used in the study are provided in Supplementary Table 1. Ethical approval for the study was obtained from the Local Research Ethics Committee of the National Hospital for Neurology and Neurosurgery.

Immunohistochemistry

7 µm thick paraffin-embedded frontal cortex sections were deparaffinized in xylene and rehydrated using graded alcohols. Immunohistochemistry for all antibodies required pretreatment in pressure cooker for 10 minutes in citrate buffer (0.1 M, pH6). Endogenous peroxidase activity was blocked (0.3 % H_2O_2 in methanol, 10 minutes) and non-specific binding with 10 % dried milk solution. Tissue sections were incubated in the relevant primary antibody in PBS-tween20 (PBS-T) for 1 hour at room temperature. The following primary antibodies were used: ADMA-GR (UCB, 1:2000), SDMA-GR (UCB, 1:50), p62 (BD Biosciences, 1:200), pTDP-43 (Cosmo Bio, 1:10,000) Tissue sections were then washed in TBS-T and incubated with the relevant biotinylated secondary antibody (DAKO, swine anti-rabbit, 1:200, or goat anti-mouse, 1:200) at room temperature for 30 minutes, prior to incubation with avidin-biotin complex (DAKO) for 30 minutes. Antibody binding was visualised by diaminobenzidine activated by H_2O_2, and cell nuclei were counterstained with Mayers haemotoxylin.

Double-label immunofluorescence

7 µm thick paraffin-embedded frontal cortex sections were stained using a rat anti-poly(GR) antibody (5H9,1:25, [15,14]) in combination with either rabbit anti- ADMA-GR (UCB, 1:2000) or SDMA-GR (UCB, 1:50). After appropriate pre-treatment, tissue sections were incubated with the primary antibodies for 1 hour at room temperature, washed in TBS-T and incubated with the secondary antibodies chicken anti-rat Alexa Fluor 488 (Thermo Fisher Scientific, 1:1000) and donkey anti-rabbit Alexa Fluor 568 (Thermo Fisher Scientific, 1:1000) for 1 hour at room temperature. Tissue sections were washed and mounted using Vectashield anti-fade mounting medium containing DAPI (Vector Laboratories) for nuclear counterstaining. Sections were viewed with a Leica DM5500B fluorescence microscope using the 40x

or 63x objectives and Leica Application Suite X software with 3D deconvolution post-processing.

Immunohistochemistry image acquisition and quantification

Frontal cortex sections stained with p62, ADMA-GR and SDMA-GR antibodies were scanned at 40x magnification using a Leica Slide Scanner SCN400 and viewed on Digital Image Hub (Leica Biosystems). Ten regions of interest were selected per case and the number of p62, ADMA-GR or SDMA-GR inclusions in each image were manually quantified to give the total number of inclusions per case. For normalisation to p62, the percentage of p62 inclusions containing ADMA-GR or SDMA-GR was calculated by dividing the total number of ADMA-GR or SDMA-GR inclusions by the total number of p62 inclusions.

Correlation analysis

All statistical analysis was performed using GraphPad Prism. All correlations were performed using Spearman rank correlation test. In all instances, a p-value of less than 0.05 was considered statistically significant.

Phase separation assay

Peptides were chemically synthesized by Pepscan (Lelystad, Netherlands), dissolved in milliQ water at 1 mM and stored at -20 °C. For droplet formation, peptides were diluted to the indicated concentrations in 100 mM K_2HPO_4/KH_2PO_4 buffer at pH7 containing 30 % polyethylene glycol (PEG; Sigma-Aldrich). OD600 of 40 µl samples was measured in clear-bottom 384 well plates (Thermo Fisher Scientific) using a SPARK Multimode microplate reader (Tecan Life Sciences). Data was analyzed using Microsoft Excel and GraphPad Prism. For imaging on plastic, droplets were incubated in plastic Cell Counter slides (Bio-Rad) and the chambers were sealed using nail varnish to prevent evaporation (described in detail in [4]). For imaging on glass, droplets were incubated in glass chambers made of microscope cover slips (Thermo Fisher Scientific), microscope slides (Thermo Fischer Scientific) and silicone spacers (Grace Bio-labs), as used previously [5]. Samples were imaged at room temperature on a Zeiss LSM 780 Meta NLO confocal microscope equipped with a 20x long-range objective. Pictures were processed with FIJI software.

Cytotoxicity assay

Primary mouse cortical neurons were dissociated into single cell suspensions from E16.5 C57BL/6 mice (Jackson Laboratory) cortices using a papain dissociation system (Worthington Biochemical Corporation). Neurons were seeded onto poly-L-lysine coated plates (0.1 % w/v) and grown in Neurobasal media (Gibco) supplemented with B-27 serum-free supplement (Gibco), GlutaMAX, and Penicillin-Streptomycin (Gibco) in a humidified incubator at 37 °C, with 5 % CO_2. Three days after seeding a half-media change was performed and peptides were added at indicated concentration for 24h. Cytotoxicity in primary neuron cultures was measured by lactose dehydrogenase (LDH) release assays (Promega, CytoTox 96® Non-Radioactive Cytotoxicity Assay), according to manufacturer's instructions. LDH readout was measured using a SPARK Multimode microplate reader (Tecan Life Sciences). Data was analyzed using Microsoft Excel and GraphPad Prism. Subsequently, the cells were fixed in 4 % formaldehyde in PBS, and stained according standard protocols. Antibodies used

were rabbit NeuN antibody (ABN78, EMD Millipore) and AlexaFluor 488 secondary antibody (Life Technologies). Samples were imaged on a Zeiss LSM 780 Meta NLO confocal microscope. Pictures were processed with FIJI software.

Poly-GR peptide uptake experiment

We labelled poly-GR20 peptides with an Alexa-488 labelling kit (Thermo-Fisher Scientific) as described before [4]. U2OS (ATCC) cells were cultured in DMEM medium (Thermo-Fisher Scientific) containing 10% FBS (Invitrogen) at 37°C and 5% CO_2, and handled according to standard procedures. Cells were seeded on glass cover slips and allowed to adhere for 24 hours. Labelled peptides were added to the cell medium at a concentration of 1 µM and incubated for 1 hour. Cells were washed before fixing with 4% PFA. Cells were imaged on a Zeiss LSM 780 Meta NLO confocal microscope. Pictures were processed and analysed with FIJI software.

Supplementary discussion

This study describes the characterisation of two novel antibodies designed to detect dimethylated poly-GR in *C9orf72* patient post-mortem tissue and is the first to demonstrate that poly-GR can be detected both in asymmetric and symmetric dimethylated forms. The ADMA-GR and SDMA-GR specific antibodies were found to label a proportion of DPR protein neuronal cytoplasmic inclusions in the frontal cortex of both *C9orf72*-FTLD and *C9orf72*-ALS cases. Symmetrically dimethylated poly-GR showed a consistently positive correlation with disease duration and age at death, indicating that this post-translational modification may have a significant effect on disease outcome.

Previous studies have suggested that this DPR protein may be post-translationally modified by the addition of methyl groups to arginine residues due to co-localisation of poly-GR with PRMT enzymes and immunoreactivity to an antibody which recognises proteins that are asymmetrically dimethylated. However, the antibodies used in the previously published studies were not specific for the poly-GR protein, making it difficult to determine whether the poly-GR protein itself was methylated, or whether it associated with other proteins that can undergo arginine methylation [3,7,19]. In this study, ELISA analysis demonstrated that novel antibodies designed to detect either symmetrically or asymmetrically modified poly-GR were specific for their respective peptide antigens, and did not show reactivity to unmethylated forms of poly-GR or to the oppositely dimethylated poly-GR antigen. The ADMA-GR antibody showed some reactivity to ADMA-PR, and the SDMA-GR antibody showed some reactivity to SDMA-PR. However, given that the antibodies had a higher affinity for the methylated poly-GR proteins, and poly-PR is rarely detected by immunohistochemistry in human post-mortem brain [9,24], it was determined that these antibodies were unlikely to identify ADMA-PR and SDMA-PR in post-mortem tissue.

Immunohistochemical assessment of the ADMA-GR and SMDA-GR antibodies showed nuclear and cytoplasmic staining in the frontal cortex of both healthy control and *C9orf72* cases. This is most likely the detection of dimethylated epitopes of other GR-containing proteins that often undergo methylation at sites with a consensus glycine/arginine rich motif, and is consistent with other studies that have investigated ADMA in human brain tissue [3,19]. The detection of inclusions, however, was specific to the *C9orf72* cases, although we cannot rule out that inclusions also contain non-DPR methylated GR-containing proteins. The number of ADMA-GR positive inclusions was consistently higher than the number of SDMA-GR positive inclusions in all *C9orf72* cases, consistent with a report showing a high degree of co-localisation between GR inclusions and an ADMA-specific antibody [19]. This is unsurprising given that asymmetric dimethylation of arginine residues is known to be the most prevalent form of methylation in physiological systems, with the monomethylated and symmetrically dimethylated forms thought to occur at levels of about 20 % to 50 % that of the asymmetrically dimethylated form [1]. ADMA-GR and SDMA-GR containing inclusions were detected in both *C9orf72*-FTLD and *C9orf72*-ALS cases. This indicates that arginine methylation of poly-GR is not specific to a clinical phenotype and cannot be used to pathologically differentiate between *C9orf72* ALS and FTLD cases, as is the case with FTLD-FUS and ALS-FUS, where methylated FUS inclusions are found in the ALS-FUS cases [10].

Double immunofluorescence demonstrated that both ADMA-GR and SDMA-GR co-localise with poly-GR, indicating that these antibodies are detecting DPR protein inclusions in the *C9orf72* cases. In agreement with the previous published studies on methylation of poly-GR inclusions, co-localisation was not observed for all poly-GR inclusions, suggesting that some inclusions may contain unmethylated or mono-methylated forms of poly-GR [3,19]. The lack of methylation in some poly-GR inclusions may reflect the dynamic reversibility of arginine methylation. Although arginine methylation was initially thought to be a permanent post-translational modification, a number of studies have emerged supporting the reversible nature of methylarginine and have identified putative arginine demethylases [2,6,21-23]. The poly-GR inclusions detected that were unmethylated may therefore reflect poly-GR proteins yet to undergo arginine methylation or that have been demethylated. The ability to be reversibly methylated could have important physical or functional consequences for the poly-GR proteins. This has been demonstrated for the FUS protein where arginine methylation alters the proteins ability to phase separate and changes its interaction with its nuclear importer [11,16].

Our observation of a significant positive correlation between SDMA-GR inclusions in the frontal cortex and both disease duration age at death indicates a potentially protective nature of SDMA-GR inclusions, as patients that have higher numbers of SDMA-GR inclusions have a longer disease duration and later age at death. Importantly, the association between SDMA-GR and disease duration or age at death remained when the DPR protein inclusion burden of each case was considered by normalising the number of SDMA-GR positive inclusions to the number of p62 positive. This indicates that these associations are specific for SDMA-GR and are not related to total DPR protein pathology in the frontal cortex.

Recent evidence showing that arginine methylation may alter a proteins ability to phase separate [11,16,17], suggests that the positive correlation of SDMA-GR in the frontal cortex with disease duration

and age at death could be hypothesised to be due to the symmetric dimethylation making poly-GR more biophysically inert. Indeed, we observed that both ADMA- and SDMA modification of poly-GR reduced its ability to phase separate through decreasing interaction strength between poly-GR molecules. As arginine promotes phase separation by both electrostatic and pi-pi interactions in this paradigm [5], methylation likely affects one or both of these interactions. Consistent with the possibility that phase separation of poly-GR contributes to its toxicity [13], these modifications also reduced poly-GR toxicity in primary neuronal cultures.

Further studies are needed to explore why only SDMA-GR and not ADMA-GR associates with longer disease duration and age at death. There is little published research into the differences in biological roles and functional consequences of ADMA and SDMA modifications, however it could be speculated that the different conformations of the dimethylated-GR may alter the biophysical properties and/or interaction of poly-GR *in vivo* in different ways.

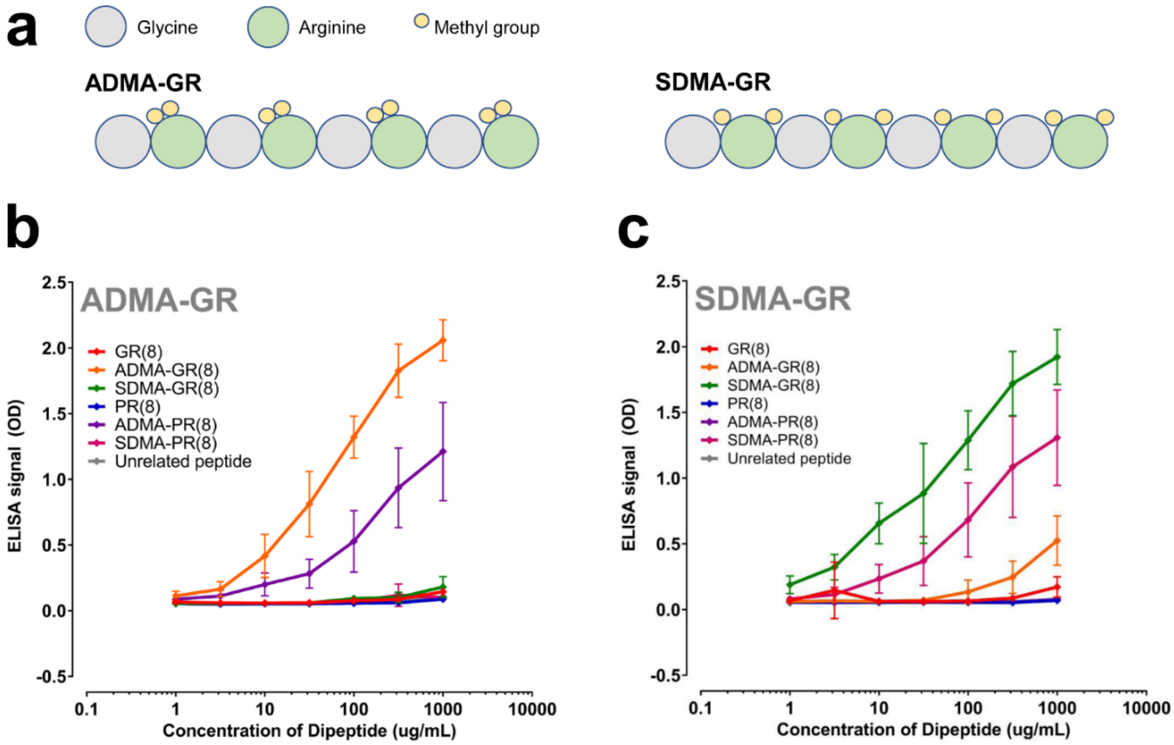

Supplementary Figure 1: Anti-GR-ADMA and GR-SDMA antibodies preferentially bind to their respective dimethylated poly-GR antigens

(**a**) Schematic representation of the ADMA-GR and SDMA-GR immunisation peptides used in antibody generation. (**b**) Specificity of the anti-ADMA-GR antibody was determined by ELISA after incubation with a range of concentrations of dimethylated or unmethylated $(DPR)_8$ peptides or an unrelated peptide. ADMA-GR shows greatest binding to the asymmetrically dimethylated poly-GR peptide, with lower binding to PR-ADMA. (**c**) Specificity of the anti-SDMA-GR antibody was determined as described in (b). SDMA-GR shows greatest binding to the symmetrically dimethylated poly-GR peptide, with lower binding to PR-SDMA. Data shown in (b) and (c) are the mean ± standard deviation of 6 individual replicates. OD = optical density.

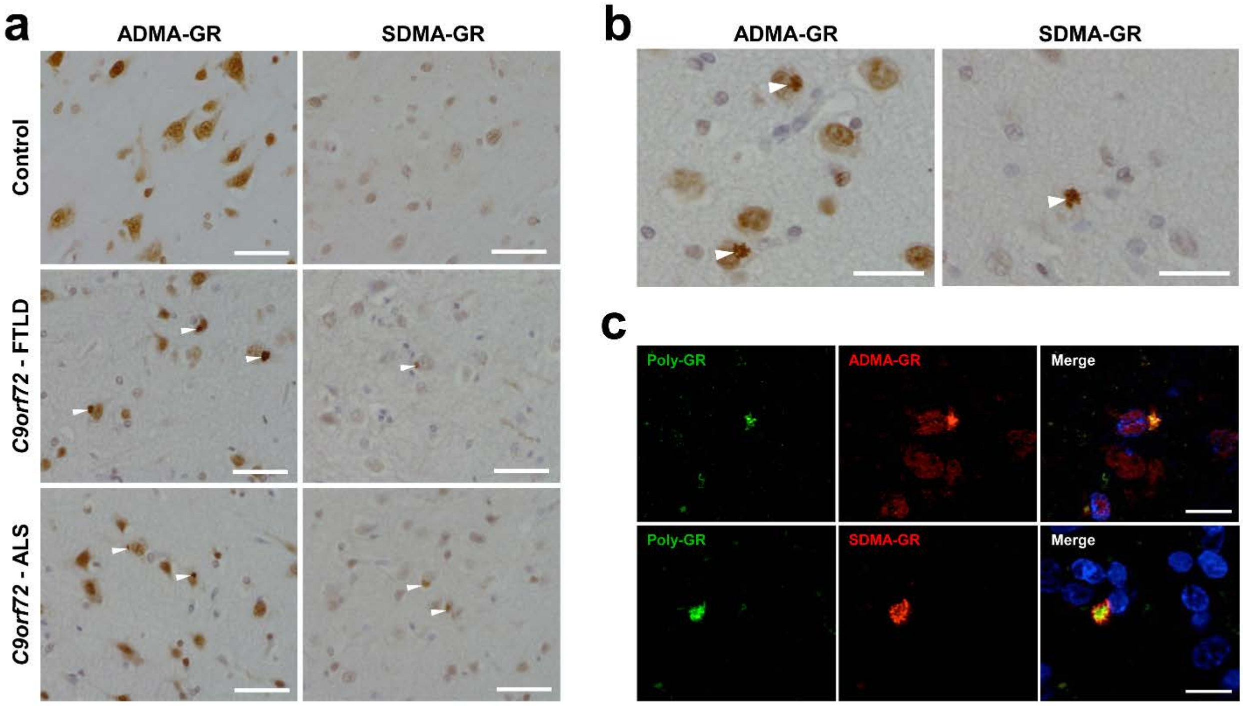

Supplementary Figure 2: ADMA-GR and SDMA-GR immunoreactive inclusion are detected in DPR inclusions in *C9orf72*-FTLD and *C9orf72*-ALS cases.

(**a**) Representative images of ADMA-GR and SDMA-GR immunohistochemical staining in the frontal cortex of a neurological normal control, *C9orf72* – FTLD case and *C9orf72* – ALS case. White arrow heads indicate neuronal cytoplasmic inclusions containing AMDA-GR or SDMA-GR. ADMA-GR and SDMA-GR antibodies did not detect dendritic poly-GR, as has previously been reported in the motor cortex of *C9orf72* ALS cases [18]. Scale bars represent 50 µm. (**b**) ADMA-GR and SDMA-GR neuronal cytoplasmic inclusions (white arrow heads) have a star-like shape; a characteristic of *C9orf72* DPR protein inclusions. Scale bars represent 20 µm. (**c**) Representative images of double immunofluorescent staining of poly-GR (green) with either ADMA-GR (red) or SDMA-GR (red) in the frontal cortex of a *C9orf72* - FTLD case. Cell nuclei are counterstained with DAPI (blue). Scale bar represents 10 µm.

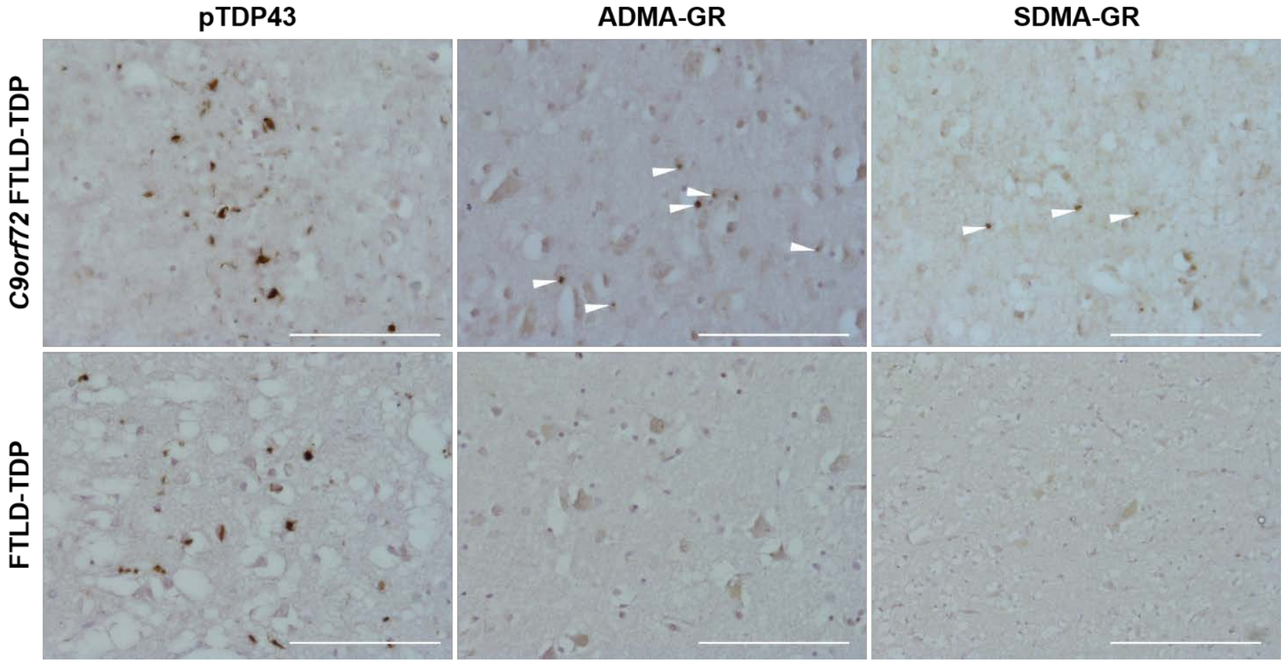

Supplementary Figure 3: ADMA-GR and SDMA-GR immunoreactive inclusions are not detected in non-*C9orf72* FTLD-TDP cases

Representative images of ADMA-GR, SDMA-GR and phosphoTDP-43 immunohistochemical staining in the frontal cortex of a *C9orf72* FTLD-TDP case and a non-*C9orf72* FTLD-TDP case. White arrow heads indicate neuronal cytoplasmic inclusions containing AMDA-GR or SDMA-GR. Scale bars represent 100 μm, n = 3 cases per condition.

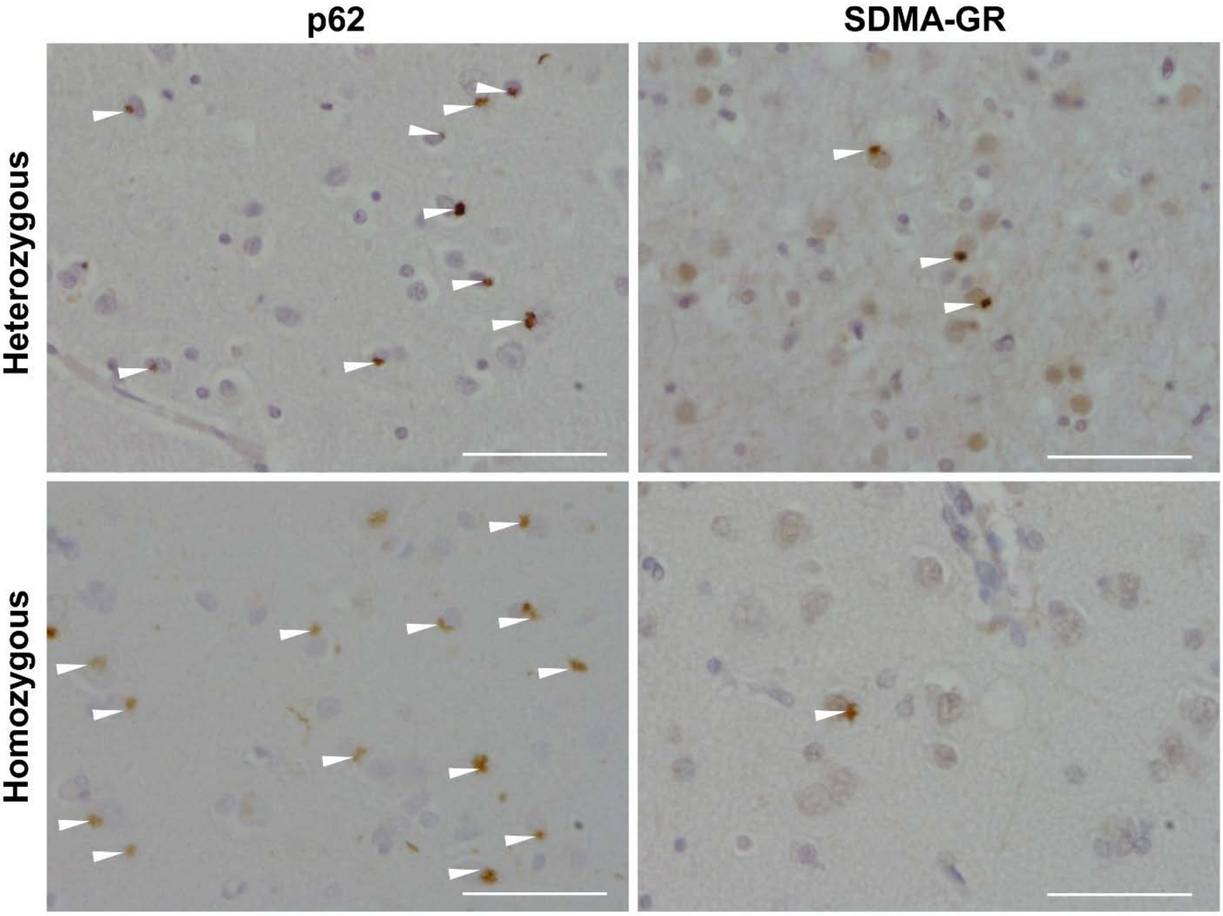

Supplementary Figure 4: A rare homozygous *C9orf72* case was observed to exhibit low levels of SDMA-GR positive inclusions despite a high p62 burden, compared to heterozygous *C9orf72* cases

Representative images of p62 and SDMA-GR immunohistochemical staining in the frontal cortex of a *C9orf72* homozygous and *C9orf72* heterozygous case. White arrow heads indicate neuronal cytoplasmic inclusions. p62 staining is shown to indicate extent of DPR protein pathology in these cases. Scale bars represent 50 μm.

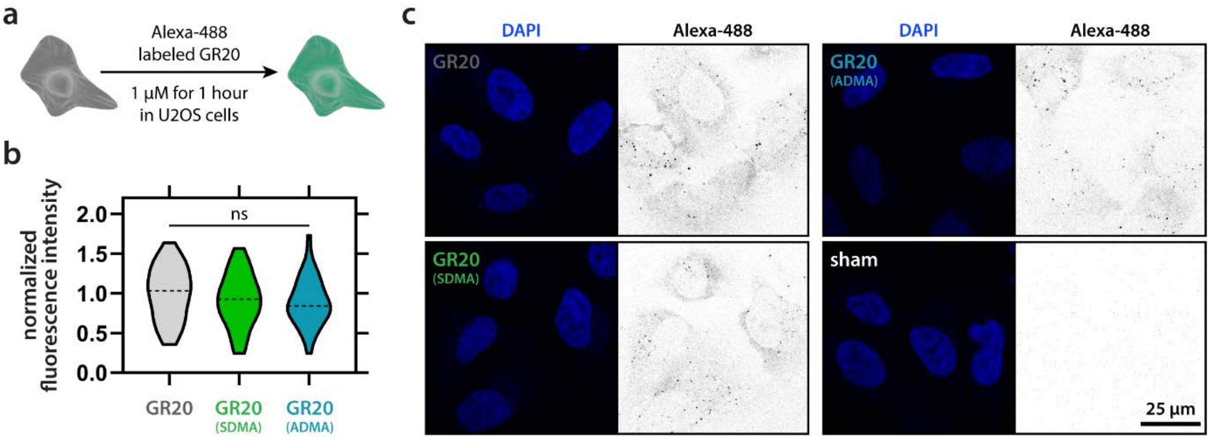

Supplementary Figure 5: Arginine dimethylation does not significantly alter peptide uptake

(**a**) We performed a modified version of a peptide uptake experiment of Alexa-488 labelled peptides, as we described previously [12]. We made use of a cancer cell line that is more resistant to GR-toxicity, and short incubation times to allow us to disentangle uptake from cellular toxicity (e.g. influx due to loss of membrane integrity). (**b**) Quantification of cytoplasmic Alexa-488 GR20 fluorescence (n = 65 cells from 2 experiments) showed that both dimethylated peptides had a non-significant ~10% decrease in the mean cytoplasmic fluorescence (one-way ANOVA, Dunnett's multiple comparisons test). (**c**) Representative pictures of U2OS cells after 1 hour of incubation with labelled peptides.

Supplementary Table 1 - Demographics of *C9orf72* cases used in this study

Case number	Brain bank	Gender	Pathological diagnosis	Mutation	Disease Duration (y)	Age of onset (y)	Age at death (y)	Post Mortem Delay (hr:min)
1	QSBB	F	ALS	*C9orf72*	5	58	63	66:30
2	QSBB	M	ALS	*C9orf72*	Unknown	Unknown	76	26:25
3	QSBB	F	FTLD-TDP type A	*C9orf72*	8	58	66	115:00
4	QSBB	M	FTLD-TDP type A	*C9orf72*	5	66	71	51:52
5	QSBB	M	FTLD-TDP type A	*C9orf72*	11	59	70	44:05
6	QSBB	F	FTLD-TDP type B	*C9orf72*	2	64	66	94:05
7	QSBB	M	FTLD-TDP type A	*C9orf72*	6	59	65	30:00
8	QSBB	F	FTLD-TDP type A	*C9orf72*	5	57	62	63:05
9	QSBB	M	FTLD-TDP type A	*C9orf72*	6	62	68	99:00
10	QSBB	M	FTLD-TDP type A	*C9orf72*	2	43	45	25:53
11	QSBB	F	FTLD-TDP type A	*C9orf72*	8	66	74	85:50
12	QSBB	M	FTLD-TDP type A	*C9orf72*	6	54	60	32:20
13	QSBB	M	FTLD-TDP type A	*C9orf72*	10	53	63	77:20
14	QSBB	F	FTLD-TDP type A	*C9orf72*	8	58	66	107:05
15	QSBB	F	FTLD-TDP type A	*C9orf72*	11	56	67	85:35
16	MBB	M	FTLD-TDP type A	*C9orf72*	9	49	58	Unknown
17	MBB	M	FTLD-TDP type A	*C9orf72*	2	63	65	95:30
18	MBB	M	FTLD-TDP type A	*C9orf72*	11	54	65	81:00
19	MBB	F	FTLD-TDP type A	*C9orf72*	5	59	64	Unknown
20	MBB	F	FTLD-TDP type B	*C9orf72*	19	51	70	48:00
21	MBB	M	FTLD-TDP type A	*C9orf72*	8	64	72	Unknown
22	MBB	M	FTLD-TDP type A	*C9orf72*	12	54	66	Unknown

23	MBB	M	ALS	*C9orf72*	Unknown	Unknown	58	35:30
24	MBB	M	ALS	*C9orf72*	7	51	58	31:30
25	MBB	M	ALS	*C9orf72*	5	60	65	Unknown
26	MBB	M	ALS	*C9orf72*	Unknown	Unknown	57	Unknown
27	MBB	M	ALS	*C9orf72*	Unknown	Unknown	45	36:00
28	MBB	F	FTLD-TDP type B	*C9orf72*	3	70	73	50:00
29	MBB	F	FTLD-TDP type B	*C9orf72*	2	63	65	50:00
30	MRCBB	M	ALS	*C9orf72*	1	69	70	38:00
31	MRCBB	M	ALS	*C9orf72*	5.5	51.5	57	22:30
32	MRCBB	M	ALS	*C9orf72*	Unknown	Unknown	51	64:00
33	MRCBB	F	ALS	*C9orf72*	3	67	70	60:00
34	MRCBB	F	ALS	*C9orf72*	3	56	59	34:30
35	MRCBB	F	ALS	*C9orf72*	5	53	58	14:00
36	MRCBB	F	ALS	*C9orf72*	4	35	39	69:30
37	MRCBB	M	ALS	*C9orf72*	2	62	64	68:00
38	QSBB	F	FTLD-TDP type A	*C9orf72*	4	83	87	69:00
39	QSBB	F	FTLD-TDP type A	*C9orf72*	6	62	68	99:45
40	QSBB	M	FTLD-TDP type A	*C9orf72*	5	57	62	92:55

QSBB = Queen Square Brain Bank, MBB = Manchester Brain Bank, MRCBB = MRC London Neurodegenerative Diseases Brain Bank, FTLD-TDP = Frontotemporal Lobar Degeneration with TDP-43 inclusions, ALS = Amyotrophic Lateral Sclerosis.

Supplementary Table 2: Spearman's rank correlation coefficients of the association between ADMA-GR and SDMA-GR in *C9orf72* frontal cortex with various clinical demographic information

Association	ADMA-GR			SDMA-GR		
	n	Spearman's r (95% CI)	*p* value	n	Spearman's r (95% CI)	*p* value
Age at onset	32	-0.098 (-0.41 to 0.27)	0.59	32	0.0051 (-0.35 to 0.36)	0.98
Age at death	37	0.24 (-0.099 to 0.53)	0.15	37	0.46 (0.15 to 0.69)	**0.0045**
Disease duration	32	0.095 (-0.27 to 0.43)	0.60	32	0.51 (0.19 to 0.74)	**0.0026**
Post-mortem delay	31	0.20 (-0.18 to 0.53)	0.28	31	0.19 (-0.19 to 0.52)	0.32

Data are Spearman's correlation coefficient r (95 % confidence interval (CI)) and p value. Significance level was set at $p < 0.05$ (two-sided). Significant p values are indicated in bold. ADMA-GR = Asymmetric dimethylated poly(GR), SDMA-GR = Symmetric dimethylated poly(GR), FTLD = Frontotemporal Lobar Degeneration, ALS = Amyotrophic Lateral Sclerosis.

Supplementary Table 3: Spearman's rank correlation coefficients of the association between the percentage of p62 inclusions containing SDMA-GR with clinical demographic information in *C9orf72* frontal cortex

**Association**	**% p62 Inclusion with SDMA-GR**		
	**n**	**Spearman's r (95% CI)**	***p* value**
Age at onset	32	0.19 (-0.18 to 0.51)	0.30
Age at death	37	0.55 (0.26 to 0.74)	**0.0005**
Disease duration	32	0.47 (0.14 to 0.711)	**0.0061**
Post-mortem delay	31	0.25 (-0.13 to 0.56)	0.18

Data are Spearman's correlation coefficient r (95 % confidence interval (CI)) and p value. Significance level was set at p < 0.05 (two-sided). Significant p values are indicated in bold. SDMA-GR = Symmetric dimethylated poly(GR), FTLD = Frontotemporal Lobar Degeneration, ALS = Amyotrophic Lateral Sclerosis.

Supplementary References

1. Bedford MT, Clarke SG (2009) Protein arginine methylation in mammals: who, what, and why. Molecular cell 33:1-13. doi:10.1016/j.molcel.2008.12.013

2. Blanc RS, Richard S (2017) Arginine Methylation: The Coming of Age. Molecular cell 65:8-24. doi:10.1016/j.molcel.2016.11.003

3. Boeynaems S, Bogaert E, Michiels E, Gijselinck I, Sieben A, Jovicic A, De Baets G, Scheveneels W, Steyaert J, Cuijt I, Verstrepen KJ, Callaerts P, Rousseau F, Schymkowitz J, Cruts M, Van Broeckhoven C, Van Damme P, Gitler AD, Robberecht W, Van Den Bosch L (2016) Drosophila screen connects nuclear transport genes to DPR pathology in c9ALS/FTD. Scientific reports 6:20877. doi:10.1038/srep20877

4. Boeynaems S, De Decker M, Tompa P, Van Den Bosch L (2017) Arginine-rich Peptides Can Actively Mediate Liquid-liquid Phase Separation. Bio-protocol 7:e2525. doi:10.21769/BioProtoc.2525

5. Boeynaems S, Holehouse AS, Weinhardt V, Kovacs D, Van Lindt J, Larabell C, Van Den Bosch L, Das R, Tompa PS, Pappu RV, Gitler AD (2019) Spontaneous driving forces give rise to protein-RNA condensates with coexisting phases and complex material properties. Proceedings of the National Academy of Sciences of the United States of America 116:7889-7898. doi:10.1073/pnas.1821038116

6. Chang B, Chen Y, Zhao Y, Bruick RK (2007) JMJD6 is a histone arginine demethylase. Science 318:444-447. doi:10.1126/science.1145801

7. Chitiprolu M, Jagow C, Tremblay V, Bondy-Chorney E, Paris G, Savard A, Palidwor G, Barry FA, Zinman L, Keith J, Rogaeva E, Robertson J, Lavallee-Adam M, Woulfe J, Couture JF, Cote J, Gibbings D (2018) A complex of C9ORF72 and p62 uses arginine methylation to eliminate stress granules by autophagy. Nature communications 9:2794. doi:10.1038/s41467-018-05273-7

8. Clargo AM, Hudson AR, Ndlovu W, Wootton RJ, Cremin LA, O'Dowd VL, Nowosad CR, Starkie DO, Shaw SP, Compson JE, White DP, MacKenzie B, Snowden JR, Newnham LE, Wright M, Stephens PE, Griffiths MR, Lawson AD, Lightwood DJ (2014) The rapid generation of recombinant functional monoclonal antibodies from individual, antigen-specific bone marrow-derived plasma cells isolated using a novel fluorescence-based method. MAbs 6:143-159. doi:10.4161/mabs.27044

9. Davidson Y, Robinson AC, Liu X, Wu D, Troakes C, Rollinson S, Masuda-Suzukake M, Suzuki G, Nonaka T, Shi J, Tian J, Hamdalla H, Ealing J, Richardson A, Jones M, Pickering-Brown S, Snowden JS, Hasegawa M, Mann DM (2016) Neurodegeneration in frontotemporal lobar degeneration and motor neurone disease associated with expansions in C9orf72 is linked to TDP-43 pathology and not associated with aggregated forms of dipeptide repeat proteins. Neuropathol Appl Neurobiol 42:242-254. doi:10.1111/nan.12292

10. Dormann D, Madl T, Valori CF, Bentmann E, Tahirovic S, Abou-Ajram C, Kremmer E, Ansorge O, Mackenzie IR, Neumann M, Haass C (2012) Arginine methylation next to the PY-NLS modulates Transportin binding and nuclear import of FUS. The EMBO journal 31:4258-4275. doi:10.1038/emboj.2012.261

11. Hofweber M, Hutten S, Bourgeois B, Spreitzer E, Niedner-Boblenz A, Schifferer M, Ruepp MD, Simons M, Niessing D, Madl T, Dormann D (2018) Phase Separation of FUS Is Suppressed by Its Nuclear Import Receptor and Arginine Methylation. Cell 173:706-719 e713. doi:10.1016/j.cell.2018.03.004

12. Kramer NJ, Haney MS, Morgens DW, Jovicic A, Couthouis J, Li A, Ousey J, Ma R, Bieri G, Tsui CK, Shi Y, Hertz NT, Tessier-Lavigne M, Ichida JK, Bassik MC, Gitler AD (2018) CRISPR-Cas9 screens in human cells and primary neurons identify modifiers of C9ORF72 dipeptide-repeat-protein toxicity. Nat Genet 50:603-612. doi:10.1038/s41588-018-0070-7

13. Lee KH, Zhang P, Kim HJ, Mitrea DM, Sarkar M, Freibaum BD, Cika J, Coughlin M, Messing J, Molliex A, Maxwell BA, Kim NC, Temirov J, Moore J, Kolaitis RM, Shaw TI, Bai B, Peng J, Kriwacki RW, Taylor JP (2016) C9orf72 Dipeptide Repeats Impair the Assembly, Dynamics, and Function of Membrane-Less Organelles. Cell 167:774-788 e717. doi:10.1016/j.cell.2016.10.002

14. Mizielinska S, Ridler CE, Balendra R, Thoeng A, Woodling NS, Grasser FA, Plagnol V, Lashley T, Partridge L, Isaacs AM (2017) Bidirectional nucleolar dysfunction in C9orf72 frontotemporal lobar degeneration. Acta neuropathologica communications 5:29. doi:10.1186/s40478-017-0432-x

15. Mori K, Arzberger T, Grasser FA, Gijselinck I, May S, Rentzsch K, Weng SM, Schludi MH, van der Zee J, Cruts M, Van Broeckhoven C, Kremmer E, Kretzschmar HA, Haass C, Edbauer D (2013) Bidirectional transcripts of the expanded C9orf72 hexanucleotide repeat are translated into aggregating dipeptide repeat proteins. Acta neuropathologica 126:881-893. doi:10.1007/s00401-013-1189-3

16. Qamar S, Wang G, Randle SJ, Ruggeri FS, Varela JA, Lin JQ, Phillips EC, Miyashita A, Williams D, Strohl F, Meadows W, Ferry R, Dardov VJ, Tartaglia GG, Farrer LA, Kaminski Schierle GS, Kaminski CF, Holt CE, Fraser PE, Schmitt-Ulms G, Klenerman D, Knowles T, Vendruscolo M, St George-Hyslop P (2018) FUS Phase Separation Is Modulated by a Molecular Chaperone and Methylation of Arginine Cation-pi Interactions. Cell 173:720-734 e715. doi:10.1016/j.cell.2018.03.056

17. Ryan VH, Dignon GL, Zerze GH, Chabata CV, Silva R, Conicella AE, Amaya J, Burke KA, Mittal J, Fawzi NL (2018) Mechanistic View of hnRNPA2 Low-Complexity Domain Structure, Interactions, and Phase Separation Altered by Mutation and Arginine Methylation. Molecular cell 69:465-479 e467. doi:10.1016/j.molcel.2017.12.022

18. Saberi S, Stauffer JE, Jiang J, Garcia SD, Taylor AE, Schulte D, Ohkubo T, Schloffman CL, Maldonado M, Baughn M, Rodriguez MJ, Pizzo D, Cleveland D, Ravits J (2018) Sense-encoded poly-GR dipeptide repeat proteins correlate to neurodegeneration and uniquely co-localize with TDP-43 in dendrites of repeat-expanded C9orf72 amyotrophic lateral sclerosis. Acta neuropathologica 135:459-474. doi:10.1007/s00401-017-1793-8

19. Sakae N, Bieniek KF, Zhang YJ, Ross K, Gendron TF, Murray ME, Rademakers R, Petrucelli L, Dickson DW (2018) Poly-GR dipeptide repeat polymers correlate with neurodegeneration and Clinicopathological subtypes in C9ORF72-related brain disease. Acta neuropathologica communications 6:63. doi:10.1186/s40478-018-0564-7

20. Tickle S, Howells L, O'Dowd V, Starkie D, Whale K, Saunders M, Lee D, Lightwood D (2015) A fully automated primary screening system for the discovery of therapeutic antibodies directly from B cells. J Biomol Screen 20:492-497. doi:10.1177/1087057114564760

21. Tsai WC, Reineke LC, Jain A, Jung SY, Lloyd RE (2017) Histone arginine demethylase JMJD6 is linked to stress granule assembly through demethylation of the stress granule-nucleating protein G3BP1. The Journal of biological chemistry 292:18886-18896. doi:10.1074/jbc.M117.800706

22. Walport LJ, Hopkinson RJ, Chowdhury R, Schiller R, Ge W, Kawamura A, Schofield CJ (2016) Arginine demethylation is catalysed by a subset of JmjC histone lysine demethylases. Nature communications 7:11974. doi:10.1038/ncomms11974

23. Wesche J, Kuhn S, Kessler BM, Salton M, Wolf A (2017) Protein arginine methylation: a prominent modification and its demethylation. Cellular and molecular life sciences : CMLS 74:3305-3315. doi:10.1007/s00018-017-2515-z

24. Xi Z, van Blitterswijk M, Zhang M, McGoldrick P, McLean JR, Yunusova Y, Knock E, Moreno D, Sato C, McKeever PM, Schneider R, Keith J, Petrescu N, Fraser P, Tartaglia MC, Baker MC, Graff-Radford NR, Boylan KB, Dickson DW, Mackenzie IR, Rademakers R, Robertson J, Zinman L, Rogaeva E (2015) Jump from pre-mutation to pathologic expansion in C9orf72. American journal of human genetics 96:962-970. doi:10.1016/j.ajhg.2015.04.016
